# Pregnancy serum concentrations of perfluorinated alkyl substances and offspring behaviour and motor development at age 5–9 years – a prospective study

**DOI:** 10.1186/1476-069X-14-2

**Published:** 2015-01-07

**Authors:** Birgit Bjerre Høyer, Cecilia Høst Ramlau-Hansen, Carsten Obel, Henning Sloth Pedersen, Agnieszka Hernik, Victor Ogniev, Bo AG Jönsson, Christian H Lindh, Lars Rylander, Anna Rignell-Hydbom, Jens Peter Bonde, Gunnar Toft

**Affiliations:** Danish Ramazzini Centre, Department of Occupational Medicine, Aarhus University Hospital, Nørrebrogade 44, building 2c, 8000 Aarhus, Denmark; Department of Public Health, Section for Epidemiology, Aarhus University, Bartholins Allé 2, 8000 Aarhus, Denmark; Department of General Practice, School of Public Health, Aarhus University, Bartholins Allé 2, 8000 Aarhus, Denmark; Primary Health Care Clinic, Postbox 570, DK-3900 Nuuk, Greenland; Department of Toxicology and Risk Assessment, National Institute of Public Health-National Institute of Hygiene, Chocimska 24, 00-791 Warsaw, Poland; Department of Social Medicine and Organization of Public Health, Kharkiv National Medical University, 61022 Kharkiv, Ukraine; Division of Occupational and Environmental Medicine, Lund University, S-221 85 Lund, Sweden; Department of Occupational and Environmental Medicine, Copenhagen University Hospital, Bispebjerg Bakke 23, 2400 Copenhagen, NV Denmark

**Keywords:** Behaviour, Child, Child development, Cohort study, Motor development, Perfluorooctanoate (PFOA), Perfluorooctane sulfonate (PFOS), Prenatal exposure, Delayed effects

## Abstract

**Background:**

In animal studies, perfluorinated alkyl substances affect growth and neuro-behavioural outcomes. Human epidemiological studies are sparse. The aim was to investigate the association between pregnancy serum concentrations of perfluorooctanoate (PFOA) and perfluorooctane sulfonate (PFOS) and offspring behaviour and motor development at 5–9 years of age.

**Methods:**

Maternal sera from the INUENDO cohort (2002–2004) comprising 1,106 mother-child pairs from Greenland, Kharkiv (Ukraine) and Warsaw (Poland) were analysed for PFOS and PFOA, using liquid-chromatography-tandem-mass-spectrometry. Exposures were grouped into country specific as well as pooled tertiles as well as being used as continuous variables for statistical analyses. Child motor development and behaviour at follow-up (2010–2012) were measured by the Developmental Coordination Disorder Questionnaire 2007 (DCDQ) and Strength and Difficulties Questionnaire (SDQ), respectively. Exposure-outcome associations were analysed by multiple logistic and linear regression analyses.

**Results:**

In the pooled analysis, odds ratio (OR) (95% confidence interval (CI)) for hyperactivity was 3.1 (1.3, 7.2) comparing children prenatally exposed to the highest PFOA tertile with those exposed to the lowest PFOA tertile. Comparing children in the highest PFOS tertile with those in the lowest PFOS tertile showed elevated but statistically non-significant OR of hyperactivity (OR (95% CI) 1.7 (0.9, 3.2)). In Greenland, elevated PFOS was associated with higher SDQ-total scores indicating more behavioural problems (β (95% CI) =1.0 (0.1, 2.0)) and elevated PFOA was associated with higher hyperactivity sub-scale scores indicating more hyperactive behaviour (β (95% CI) = 0.5 (0.1, 0.9)). Prenatal PFOS and PFOA exposures were not associated with motor difficulties.

**Conclusions:**

Prenatal exposure to PFOS and PFOA may have a small to moderate effect on children’s neuro-behavioural development, specifically in terms of hyperactive behaviour. The associations were strongest in Greenland where exposure contrast is largest.

**Electronic supplementary material:**

The online version of this article (doi:10.1186/1476-069X-14-2) contains supplementary material, which is available to authorized users.

## Introduction

Perfluorinated alkyl substances (PFAS) are man-made persistent chemicals, which are being widely used due to their water and oil-repellent properties. Biomonitoring studies have shown that they are found globally in a variety of living organisms, including humans [1–4]. Exposure pathways are mainly through diet and drinking water [5, 6]. Perfluorooctanoate (PFOA) and perfluorooctane sulfonate (PFOS) are widespread in the environment in spite of a phase out since year 2000. They have long serum half-lives in humans [7] and are known to cross the placenta [8], which is worrying as fetal and early life is considered to be the vulnerable period of normal brain development [9].

Some of the most prevalent neuro-developmental disorders are developmental coordination disorder and attention deficit hyperactivity disorder (ADHD) and they may be related through common causal pathways [10]. The world prevalence of developmental coordination disorder is 5-6% [11–13] and the world prevalence of ADHD is 5-10% [14]. The disorders are related to various predictors such as fetal alcohol exposure [15] and fetal smoking exposure [16] although newer studies report little evidence of a causal relation between prenatal smoking exposure and development of ADHD [17]. Twin studies of ADHD suggest a high level of heritability, but fetal environmental pollutant exposures are also likely to play a role [18].

Animal studies suggest associations between prenatal exposure to PFOS and PFOA and neuro-behavioural deficits [19–21]. Human observational studies on the relation between PFAS exposures and behaviour are sparse and results are inconsistent, which may be due to different exposure levels and study designs. One cross-sectional study reported a positive association between PFOS and PFOA and ADHD [22], while another study observed an inverse J-shaped association between PFOA and ADHD across quartiles of exposure [23], indicating elevated risk of ADHD among those exposed to medium exposure level compared to low exposure level. No association was observed between relatively high fetal PFOS and PFOA exposure and behaviour at 7 years of age [24], while another follow-up study with lower PFAS exposures observed a relation between higher levels of PFOS (but not PFOA) and worse gross motor function in 2-year old children [25].

The present longitudinal study with a relatively large number of participants and relatively large exposure spans investigated the associations between pregnancy concentrations of PFOS and PFOA at background exposure levels and motor development and behaviour in 5-9-year old children.

## Methods

### Study population and data collection

During the period from May 2002 to February 2004, 1,441 pregnant women from Greenland, Warsaw (Poland) and Kharkiv (Ukraine) were enrolled in the INUENDO (Biopersistent organochlorines in diet and human fertility) birth cohort from antenatal health care clinics and provided a blood sample at any stage of pregnancy. To be eligible for the study, the woman had to be born in the country of study, be pregnant and at least 18 years of age. At baseline, 2,478 women were eligible in Ukraine of whom 612 (24.7%) participated, and non-participants were slightly younger than participants but were otherwise similar. In Greenland, 665 were eligible and 571 (85.9%) participated, and participants and non-participants were very similar. In Poland, 690 were eligible and 258 (37.4%) participated. Unfortunately, we have no information available for the non-participants from Poland, limiting our ability to compare participants and non-participants. Further details on the baseline study population are available elsewhere [26]. A follow-up was conducted from January 2010 to May 2012, when the children were between 5 and 9 years old. Parents or guardians responded to questions concerning lifestyle, motor development, behaviour and other characteristics in a face-to-face interview or by filling in a questionnaire themselves.

At follow-up, a total of 1,113 mother-child-pairs (singleton births) with measured exposure information participated of the 1,441 pregnant women enrolled at baseline. After exclusion of mother-child-pairs with missing information on all 15 DCDQ items (n = 7) the study population consisted of 1,106 children, distributed between Greenland (n = 526 (47.6%)), Poland (n = 89 (8.0%)) and Ukraine (n = 491 (44.4%)).

### Determination of PFOS and PFOA and cotinine

Plasma concentrations of PFOA, PFOS and cotinine were analysed, using liquid chromatography-tandem-mass-spectrometry (LC/MS/MS) at The Department of Occupational and Environmental Medicine in Lund, Sweden. A detailed description of the method for PFOS and PFOA is presented elsewhere [27]. Cotinine was analysed by the same method. Briefly, aliquots of 100 μl serum were added 25 μl of a water:acetonitrile (50:50) solution containing labelled internal standards. Proteins were precipitated with acetonitrile and vigorously shaking for 30 minutes. The samples were then centrifuged and the supernatant analyzed using a LC (UFLCXR, SHIMADZU Corporation, Kyoto, Japan) connected to a hybrid triple quadrupole linear ion trap mass spectrometer (QTRAP 5500, AB Sciex, Foster City, CA, USA). All samples were above limits of detection, which were 0.2 ng/ml and 0.04 ng/ml for PFOS and PFOA, respectively. Coefficient of variation of duplicate samples worked-up and analyzed on different days were 9% for PFOS and 11% for PFOA. The analyses of PFOA and PFOS are part of the Round Robin inter-comparison program (Professor Dr. Med. Hans Drexler, Institute and Out-patient Clinic for Occupational-, Social- and Environmental Medicine, University of Erlangen-Nuremberg, Germany) with results within the tolerance limits.

### Assessment of child behaviour and motor abilities and covariate data

Behaviour was assessed using the parent version of the standardized questionnaire “The Strength and Difficulties Questionnaire” (SDQ), comprising 25 items on five scales (emotional, conduct, hyperactivity, peer and pro-social behaviour) [28]. SDQ is a screening tool used to identify common mental disorders in children 4 to 16 years of age. The items were coded 0 “not true”, 1 “somewhat true” or 2 “certainly true”. Each scale had a summed score ranging from 0 to 10. A SDQ-total score was calculated by summing four of the scales (emotional, conduct, hyperactivity and peer) with a score range of 0 to 40. Cut-offs on the SDQ were set according to standard (SDQ-total:0 to 13 = normal, 14 to16 = borderline and 17 to 40 = abnormal; Emotional symptoms: 0 to 5 = normal, 6 = borderline and 7 to 10 = abnormal; Conduct problems: 0 to 3 = normal, 4 = borderline and 5 to 10 = abnormal; Hyperactivity: 0 to 5 = normal, 6 = borderline and 7 to 10 = abnormal; Peer problems: 0 to 3 = normal, 4 to 5 = borderline and 6 to 10 = abnormal; Pro-social behaviour: 6 to 10 = normal, 5 = borderline and 1 to 4 = abnormal) [29]. When outcomes were dichotomised, the cut-offs were normal/borderline versus abnormal. In all scales, except the pro-social subscale, a high score indicated problems.

Parents were asked to assess their child’s behaviour during the past six months.

To evaluate the motor development, parents compared their child’s motor abilities to that of his or her peers using “Developmental Coordination Disorder Questionnaire 2007” (DCDQ). DCDQ is a screening tool to help identify motor difficulties in children 5-15-years of age, comprising 15 items on a 5-point Likert scale with a total score range of 15 to 75 [30]. A low score indicates problems. Only continuous DCDQ scores were used in this study as scores in the three populations were heterogeneous and the validity of DCDQ has not been examined specifically in the countries where the present study takes place.

Covariate data were collected from the pregnancy and follow-up questionnaires and included information about e.g. lifestyle, health and personal characteristics.

### Statistical analysis

#### Missing information

The number of missing values on behaviour, motor development and covariates ranged from 0 to 55%. One of the 15 items (question 12; “your child learns new motor tasks easily”) was erroneously lacking in the Greenlandic version of the DCDQ (but not in the Danish version of the questionnaire used for some participants in Greenland), which caused 55% missing of the DCDQ in Greenland, as all items are needed to generate a total score. In the Greenlandic population, the median of missing answers of the DCDQ were 2%, and 85% had answered at least 14 of 15 items of the DCDQ. To increase power and overcome the risk of introducing selection bias by analysing only the complete case dataset, we used chained multiple imputation, allowing us to maintain participants with incomplete data [31]. Briefly, multiple different imputed datasets (*m* > 1) are created, and a set of random plausible values replace each missing value, based on known subject characteristics and other predictors in the complete dataset. This incorporates an appropriate variability across the *m* datasets. The new *m* complete datasets are analysed, producing a single set of results accounting for the variability of the missing data [31].

We generated 100 imputed datasets in a combined imputation that included all three countries. The predictors were: PFOA, PFOS, the 15 items of the DCDQ, the 5 subscales of the SDQ, maternal cotinine level during pregnancy, maternal alcohol consumption before conception, maternal educational level, maternal age at pregnancy, birth weight, gestational age at birth, gestational age at blood sampling, parity, breastfeeding duration, child age at follow-up and child sex. The robustness of the imputation model was examined creating fewer (*m* = 20) and more (*m* = 150) datasets and using less and more predictors, and furthermore, complete case analyses were performed as sensitivity analyses.

### Data analysis

A non-response analysis was performed to check for inconsistencies between responders (n = 1,113) and non-responders (n = 328). Spearman’s rank correlation was used to assess the correlation between maternal pregnancy levels of PFOS and PFOA. We used logistic regression models in the primary analyses of PFOS and PFOA levels and behavioural problems (abnormal SDQ-total and sub-scale scores) and linear regression in the investigation of PFOS and PFOA exposure and motor development. Exposures were *a priori* decided to be categorized into tertiles per country as well as pooled, using the lowest tertile as reference group. Moreover, trends in exposure-outcome associations were further explored, using continuous, natural logarithm transformed exposures in linear regression models. To increase power, the associations between exposures and behavioural outcomes were also examined using linear regression models with exposures categorized into tertiles as well as a continuous variable. All estimates were adjusted for the most important potential confounders among the available data, which were identified *a priori* and included maternal cotinine level during pregnancy (serum cotinine ≤10 (non-smoker)/ >10 ng/ ml (smoker)); maternal alcohol consumption at conception (0, <7, ≥7 drinks per week); maternal age at pregnancy (continuous); child sex and gestational age at blood-sampling (continuous) [15, 16, 32, 33]. In addition, we explored possible interactions between the exposures and sex and exposures and country, adding interaction terms to the model. In a sensitivity analysis of the SDQ, we used the top 10 percentile cut-off from this sample as a marker of behavioural problems instead of the standard cut-offs suggested by Youth in Mind [29] as also used in another study [24]. Furthermore, duration of breastfeeding was added as covariate to adjust for postnatal PFAS exposures. Complete case results are presented as supplementary material.

All analyses were performed stratified by population as well as combined (adjusted for population). Not all analyses could be performed in the Polish dataset since only very few cases were present. A p-value less than 0.05 was considered statistically significant. Statistical analyses were performed using the Stata statistical package (version 12.1, StataCorp, College Station, Texas, USA).

### Ethics

The study was approved by local ethical committees; Polish Bioethical Committee (approval no. 6/2002 of 3.07.2002), Ethical Committee for Human Research in Greenland (approval no. 2010–13) and the Commission on Ethics and Bioethics Kharkiv National Medical University in Ukraine (protocol number 7, October 7 2009). All participating parents signed informed consent.

## Results

The non-response analysis showed no differences between responders and non-responders at follow-up concerning exposure levels, maternal educational level, pregnancy smoking status and maternal age at baseline (data not shown). The median (10–90 percentile) age of the children at follow-up was 8 (7 to 9) years in Greenland compared to 7 (7 to 8) and 8 (7 to 8) years in Ukraine and Poland, respectively. Personal characteristics of the study population are presented in Table 1.

**Table 1:** **Characteristics of mothers and their children according to country**

Characteristics	Combined	Greenland	Ukraine	Poland
	(N = 1,106)	(n = 526)	(n = 491)	(n = 89)
**Outcome**				
DCDQ-score (points) ^a^	52 (40–69)	63 (51–75)	46 (37–55)	64 (52–73)
Missing, n (%)	298 (27)	289 (55)	6 (1)	3 (3)
SDQ-score (points) ^a^	7 (2–15)	7 (2–14)	8 (4–14)	8 (2–15)
Missing, n (%)	7 (1)	6 (1)	0 (0)	1 (1)
SDQ, n (%)				
Normal	954 (86)	455 (88)	424 (86)	75 (85)
Borderline	82 (7)	34 (6)	40 (8)	8 (9)
Abnormal	63 (6)	31 (6)	27 (6)	5 (6)
Hyperactivity, n (%)				
Normal	967 (87)	477 (91)	425 (86)	65 (73)
Borderline	70 (6)	20 (4)	42 (9)	8 (9)
Abnormal	62 (6)	23 (4)	24 (5)	15 (17)
Missing, n (%)	7 (1)	6 (1)	0 (0)	1 (1)
**Maternal characteristics**				
Maternal age at pregnancy, years	26 (20–35)	26 (20–37)	24 (20–32)	29 (26–34)
Missing, n (%)	62 (6)	38 (7)	24 (5)	0 (0)
Maternal pre-pregnancy BMI, kg/m^2 a^	22 (19–28)	24 (20–30)	21 (18–26)	21 (19–24)
Parity, no. (%)				
0	650 (59)	167 (33)	399 (81)	84 (94)
1 or more	432 (39)	335 (67)	92 (19)	5 (6)
Missing, n (%)	24 (2)	24 (5)	0 (0)	0 (0)
Maternal cotinine level during pregnancy, no. (%)				
>10 ng/ml - smoker	374 (34)	296 (56)	76 (15)	2 (2)
≤10 ng/ml – non-smoker	732 (66)	230 (44)	415 (85)	87 (98)
Maternal alcohol consumption at conception, no. (%)				
≤7 drinks/week	1,040 (94)	465 (88)	491 (100)	84 (94)
>7 drinks/week	66 (6)	61 (12)	0 (0)	5 (6)
Missing ^b^	582 (500)	261 (50)	307 (63)	14 (16)
Maternal educational level, left school at age, no. (%)				
<18 years	356 (32)	217 (46)	139 (28)	0 (0)
≥18 years	621 (56)	249 (54)	293 (68)	79 (100)
Missing, n (%)	129 (12)	60 (11)	59 (12)	10 (11)
**Child characteristics**				
Sex, no. (%)				
Male	595 (54)	282 (53)	260 (53)	53 (60)
Female	505 (45)	241 (46)	228 (46)	36 (40)
Missing, n (%)	6 (1)	3 (1)	3 (1)	0 (0)
Total breastfeeding duration, no. (%)				
0 months	62 (6)	17 (4)	43 (9)	2 (2)
<6 months	302 (27)	120 (25)	163 (33)	19 (22)
6–12 months	334 (30)	124 (26)	175 (36)	35 (39)
>12 months	354 (32)	214 (45)	107 (22)	33 (37)
Missing, n (%)	54 (5)	51 (10)	3 (1)	0 (0)
Gestational age, no. (%)				
≥37 weeks	1,043 (95)	499 (95)	479 (98)	65 (94)
<37 weeks	37 (3)	24 (5)	9 (2)	4 (6)
Missing, n (%)	26 (2)	3 (1)	3 (1)	20 (22)
Gestational age at blood sample, weeks ^a^	39 (38–41)	25 (13–37)	23 (9–40)	33 (27–37)
Birth weight, grams ^a^	3,430 (2,800-4,130)	3,600 (2,840-4,370)	3,285 (2,800-3,800)	3,460 (2,880-4,000)
Age at follow-up, years ^a^	7 (7–9)	8 (7–9)	7 (7–8)	8 (7–8)
Missing, n (%)	58 (5)	53 (10)	5 (1)	0 (0)

BMI, body mass index; DCDQ, developmental coordination disorder questionnaire; PFOA, perfluorooctanoate; PFOS, perfluorooctane sulfonate; SDQ, strength and difficulties questionnaire. All percentages are based on complete-case data. ^a^ Median (10^th^-90^th^ percentile). ^b^ Missing alcohol information was set to 0 intake.

In comparison with women from Greenland, women from Poland were older, had lower body mass index (BMI), more often expected their first child, were non-smokers and better educated. Women from Ukraine were younger at recruitment, had lower BMI, were more often non-smokers and drank less alcohol compared to women from Greenland (Table 1). The level of missing data is presented in Table 1, showing highest level of missing data on the DCDQ in Greenland.

The median serum concentrations of PFOS and PFOA are presented in Table 2 alongside the exposure tertiles across the three countries and pooled. Median PFOS exposure levels were highest in Greenland (median (10^th^–90^th^ percentile) PFOS 20.3 ng/ml (12.0, 37.0)), which is approximately 4 times higher than the median PFOS exposure level in Ukraine. The median PFOA levels were highest in Poland (median (10^th^-90^th^ percentile) 2.7 ng/ml (1.5, 4.3)).

**Table 2:** **Range (ng/ml) of PFOS/PFOA plasma concentrations, by tertile, in pregnant women**

Exposure	Tertile	Combined	Greenland	Ukraine	Poland
		(N = 1,106)	(n = 526)	(n = 491)	(n = 89)
**Pregnancy PFOS**	Median (10^th^-90^th^ percentile)	10.0 (3.6, 27,4)	20.3 (12.0-37.0)	5.0 (2.9-8.2)	8.0 (5.3-12.7)
	Low tertile	0.7-6.2	4.1-16.8	0.7-4.2	2.5-7.0
	Medium tertile	6.2-16.6	16.8-23.9	4.2-5.9	7.1-9.6
	High tertile	16.6-87.3	23.9-87.3	5.9-18.1	9.7-21.3
**Pregnancy PFOA**	Median (10^th^-90^th^ percentile)	1.4 (0.7, 3.0)	1.8 (1.0-3.1)	1.0 (0.5-1.7)	2.7 (1.5-4.3)
	Low tertile	0.2-1.1	0.5-1.5	0.2-0.8	1.0-2.2
	Medium tertile	1.1-1.9	1.5-2.2	0.8-1.1	2.2-3.1
	High tertile	1.9-9.8	2.2-5.1	1.1-9.8	3.1-9.8

PFOA, perfluorooctanoate; PFOS, perfluorooctane sulfonate.

The Spearman’s correlation coefficient between maternal pregnancy levels of PFOS and PFOA was 0.5 in Greenland, 0.6 in Poland and 0.5 in Ukraine.

In Table 3, results of the linear regression analyses between PFOS and PFOA and motor skills (DCDQ) are presented. In the combined analysis of all three populations, PFOS and PFOA were associated with a minor decrease in DCDQ score. All associations between PFOS and PFOA and motor development were clinically minor and statistically non-significant. No interaction was observed between exposures and sex and exposures and country.

**Table 3:** **Associations** ^**a**^ **between pregnancy levels of PFOS/PFOA (ng/ml) and offspring DCDQ-score** ^**b**^

Exposure		Combined ^d^ (N = 1,106)	Greenland (n = 526)	Ukraine (n = 491)	Poland (n = 89)
		β (95% CI)	β (95% CI)	β (95% CI)	β (95% CI)
**PFOS**	Low	ref	ref	ref	ref
	Medium	−0.4 (−1.9, 1.2)	−1.2 (−3.0, 0.7)	1.4 (−0.2, 3.1)	−0.3 (−6.0, 5.3)
	High	−1.7 (−3.8, 0.5)	−0.7 (−2.6, 1.2)	0.6 (−1.1, 2.2)	−2.1 (−7.8, 3.5)
	Continuous^c^	−0.1 (−1.2, 1.1)	−0.2 (−1.9, 1.4)	0.5 (−1.0, 2.0)	−2.4 (−9.1, 4.3)
**PFOA**	Low	ref	ref	ref	ref
	Medium	−0.6 (−1.9, 0.7)	−1.2 (−3.0, 0.7)	−0.2 (−1.8, 1.4)	1.5 (−4.1, 7.1)
	High	−0.4 (−1.9, 1.1)	−0.1 (−2.0, 1.7)	−1.2 (−2.8, 0.5)	−3.7 (−9.3, 1.9)
	Continuous^c^	−0.2 (−1.2, 0.9)	0.8 (−0.8, 2.5)	−0.6 (−1.9, 0.8)	−2.7 (−8.3, 2.8)

CI, confidence interval; DCDQ, developmental coordination disorder questionnaire; PFOA, perfluorooctanoate; PFOS, perfluorooctane sulfonate; Ref, referece group.

^a^Adjusted for: maternal cotinine level during pregnancy, maternal alcohol consumption at conception, maternal age at pregnancy, gestational age at blood-sampling and child sex. ^b^DCDQ score range from 15 to 75 (low scores indicate motor problems). ^c^β = the change in score according to one natural-log unit increase in PFOA and PFOS. ^d^additionally adjusted for country.

The associations between PFOA and PFOS and behavioural problems (continuous SDQ-total and hyperactivity) are presented in Table 4. In the combined analyses of all three countries, high PFOA and PFOS exposures compared with low exposures were associated with a 0.5 point higher hyperactivity scores (more hyperactive behaviour). Higher PFOS levels were associated with higher SDQ-total scores (more behavioural problems) in Greenland (Table 4). No associations were observed in Poland and Ukraine. PFOA was positively associated with hyperactive scores in Greenland (β (95% CI) = 0.5 (0.1, 0.9)) but not in Poland and Ukraine. PFOA was not associated with SDQ-total in any of the populations. There was no evidence of interaction between exposures and sex or between exposures and country.

**Table 4:** **Associations** ^**a**^ **between pregnancy levels of PFOS/PFOA (ng/ml) and offspring SDQ-total and hyperactivity score**

			Combined (N = 1,106) ^e^		Greenland (n = 526)		Ukraine (n = 491)		Poland (n = 89)	
Scale (score range)			Difference	95% CI	Difference	95% CI	Difference	95% CI	Difference	95% CI
**SDQ (0–40)** ^**b**^	**PFOA**	Low	Ref	-	Ref	-	Ref	-	Ref	-
		Medium	0.3	−0.5, 1.0	0.7	−0.4, 1.7	−0.9	−1.9, 0.1	0.8	−1.9, 3.4
		High	0.7	−0.2, 1.5	0.5	−0.6, 1.5	−0.3	−1.3, 0.7	1.8	−0.8, 4.5
		Continuous^d^	0.1	−0.5, 0.7	0.3	−0.6, 1.3	−0.5	−1.3, 0.4	2.1	−0.4, 4.7
**Hyperactivity (0–10)** ^**c**^		Low	Ref	-	Ref	-	Ref	-	Ref	-
		Medium	0.1	−0.3, 0.4	0.5	0.0, 1.0	−0.3	−0.7, 0.1	1.1	−0.4, 2.6
		High	0.5	0.1, 0.9	0.6	0.1, 1.1	−0.2	−0.6, 0.2	0.9	−0.6, 2.4
		Continuous^d^	0.3	0.0, 0.5	0.5	0.1, 0.9	−0.1	−0.5, 0.2	1.2	−0.3, 2.6
**SDQ (0–40)** ^**b**^	**PFOS**	Low	Ref	-	Ref	-	Ref	-	Ref	-
		Medium	0.1	−1.0, 0.8	1.3	0.3, 2.4	−1.0	−2.0, 0.0	−0.1	−2.7, 2.5
		High	1.1	−0.1, 2.3	1.1	0.1, 2.2	−1.0	−2.2-0.0	2.0	−0.6, 4.6
		Continuous^d^	0.3	−0.3, 1.0	1.0	0.1, 2.0	−1.0	−1.6, 0.2	2.6	−0.5, 5.7
**Hyperactivity (0–10)** ^**c**^		Low	Ref	-	Ref	-	Ref	-	Ref	-
		Medium	0.1	−0.3-0.5	0.4	−0.1, 0.8	−0.2	−0.6, 0.2	0.9	−0.5, 2.4
		High	0.5	0.0, 1.0	0.3	−0.2, 0.7	−0.1	−0.5, 0.3	1.3	−0.1, 2.8
		Continuous^d^	0.2	−0.1, 0.5	0.3	−0.1, 0.7	−0.1	−0.4, 0.3	1.5	−0.3, 3.2

CI, confidence interval; PFOA, perfluorooctanoate; PFOS, perfluorooctane sulfonate; Ref, reference group; SDQ, strength and difficulties questionnaire.

^a^Adjusted for: maternal cotinine level during pregnancy, maternal alcohol consumption at conception, maternal age at pregnancy, gestational age at blood-sampling and child sex. ^b^High scores indicate behavioural problems. ^c^High scores indicate more hyperactive behaviour. ^d^β = the change in score according to one natural-log unit increase in PFOA and PFOS. ^e^Additionally adjusted for country.

Table 5 presents results of the adjusted logistic regression analyses of the associations between PFOS and PFOA and dichotomized (normal/borderline versus abnormal) SDQ-total and hyperactivity. In the combined analysis, OR (95% CI) for abnormal SDQ-total was 2.7 (1.2, 6.3) comparing highest PFOA tertile to lowest PFOA tertile. In Greenland, the adjusted OR for hyperactive behaviour was higher comparing medium and high PFOA exposures to low PFOA exposure, although the 95% CIs were wide (OR (95% CI) = 5.4 (1.1, 25.6) and 6.3 (1.3, 30.1), respectively). In Ukraine, no associations were observed. PFOS was not associated with abnormal SDQ-total in any of the analyses.

**Table 5:** **Associations** ^**a**^ **between pregnancy levels of PFOS/PFOA (ng/ml) and offspring behavioural and hyperactivity problems**

Scale	Combined ^e^	Greenland	Ukraine	Poland
	Adjusted OR (95% CI)	Adjusted OR (95% CI)	Adjusted OR (95% CI)	Adjusted OR (95% CI)
	(N = 1,106)	(n = 526)	(n = 491)	(n = 89)
**SDQ-total** ^**b**^				
PFOS				
Low	1.0 (ref)	1.0 (ref)	1.0 (ref)	1.0 (ref)
Medium	1.1 (0.5, 2.5)	1.7 (0.7, 4.0)	0.6 (0.2, 1.6)	-
High	1.5 (0.5, 4.8)	0.8 (0.3, 2.2)	0.6 (0.2, 1.5)	-
Continuous^d^	1.1 (0.6, 2.0)	0.9 (0.4, 2.1)	1.0 (0.4, 2.5)	-
PFOA				
Low	1.0 (ref)	1.0 (ref)	1.0 (ref)	1.0 (ref)
Medium	2.0 (1.0, 4.2)	2.7 (1.0, 7.5)	1.3 (0.5, 3.5)	-
High	2.7 (1.2, 6.3)	1.9 (0.7, 5.4)	1.5 (0.6, 4.2)	-
Continuous^d^	1.5 (0.9, 2.6)	2.1 (0.8, 5.2)	1.0 (0.4, 2.3)	-
**Hyperactivity** ^**c**^				
PFOS				
Low	1.0 (ref)	1.0 (ref)	1.0 (ref)	-
Medium	1.2 (0.5, 2.5)	1.1 (0.4, 3.4)	1.2 (0.4, 3.3)	-
High	1.4 (0.4, 4.9)	1.3 (0.4, 3.9)	1.4 (0.5, 3.9)	-
Continuous^d^	1.7 (0.9, 3.2)	1.9 (0.7, 4.8)	1.3 (0.5, 3.4)	-
PFOA				
Low	1.0 (ref)	1.0 (ref)	1.0 (ref)	-
Medium	0.8 (0.4, 2.0)	5.4 (1.1, 25.6)	0.8 (0.3, 2.3)	-
High	3.1 (1.3, 7.2)	6.3 (1.3, 30.1)	0.9 (0.3, 2.4)	-
Continuous^d^	1.6 (0.9, 2.8)	3.6 (1.2, 3.7)	0.8 (0.3, 1.9)	-

CI, confidence interval; OR, odds ratio; PFOA, perfluorooctanoate; PFOS, perfluorooctane sulfonate; SDQ, strength and difficulties questionnaire; Ref, reference group.

^a^Adjusted for maternal cotinine level during pregnancy, maternal alcohol consumption at conception, child sex, maternal age at pregnancy and gestational age at blood sampling.

^b^SDQ cut-offs: normal and borderline (0 to 16) versus abnormal (17 to 40).

^c^Hyperactivity cut-offs: normal and borderline (0 to 6) versus (7 to 10).

^d^The change in OR according to one natural logarithm increase in exposures.

^e^Additionally adjusted for country.

Few associations were found for the other SDQ sub-scales (emotional, conduct, peer and pro-social) (See Additional file 1). The OR for abnormal pro-social behaviour was also above 1 in Ukraine but not in Greenland.

Results from complete-case analyses were generally similar to the imputation-based analyses presented above (See Additional files 2, 3 and 4).

In the sensitivity analysis using top ten percentile cut-offs results were somewhat attenuated but the overall pattern did not change (Additional file 5). Adding duration of breastfeeding as a covariate to the models did not change direction or significance of results.

Results of sensitivity analyses of smaller and larger imputation models showed similar results, indicating a robust imputation model.

## Discussion

We observed a small to moderate positive association between pregnancy levels of PFOA and child hyperactive behaviour in the total sample, which was mainly attributable to associations in Greenland. In Greenland, PFOS was associated with behavioural problems (SDQ-total) (not in Poland and Ukraine), whereas PFOA was associated with hyperactivity. Only minor associations appeared in relation to other SDQ sub-scales and none to motor development, as measured using DCDQ.

The mechanism related to the association between prenatal PFAS exposure and behavioural and motor problem is unclear. However, an association between prenatal PFOS exposure and thyroid hormone disruption has been reported in mice [34]. In humans, higher pregnancy levels of PFOS have been associated with higher pregnancy levels of thyroid-stimulating hormone (TSH) [35], and higher pregnancy levels of some PFAS (perfluorononanoic acid (PFNA), perfluoroundecanoic acid (PFUnDA) and perfluorododecanoic acid (PFDoDA) have been associated with lower infant cord levels of triiodothyronine (T3) and total free thyroxine (T4) [36]. Since thyroid hormones are essential for fetal and early life neurologic development, a disruption by prenatal PFAS exposures could possibly impair healthy neurodevelopment.

The results of epidemiological studies examining the neurodevelopmental effects of PFAS are few and inconclusive. Fei et al. reported no associations between high pregnancy levels of PFOA (median (inter-quartile-range): 5.4 (4.0-7.1 ng/ml)) and PFOS (34.4 (26.6-44.5 ng/ml)) and developmental coordination disorder (assessed by DCDQ’07) at 7 years among a sub-sample of 537 participants from the Danish National Birth Cohort [24], which is in accordance with our results. In the same study, no associations were observed between PFOS and PFOA and SDQ-total or any of the SDQ sub-scales, using top 10 percentile cut-offs [24]. This is in accordance with our sensitivity analysis using top 10 percentile cut-off, whereas we observed associations between PFOA and hyperactivity in Greenland and in the combined analysis of the three countries in our main analysis using standard cut-offs. Furthermore, we observed a minor positive association between PFOS and SDQ-total in the combined analysis, however, statistically non-significant, whereas the country specific results were more heterogeneous. In the present study, we used the standard cut-offs suggested by Youth in Mind [29] and 804 of the 1,106 included children were older than 7 years, which may explain the difference in our results.

A small Taiwanese birth cohort study with exposure levels equivalent to exposure levels in this study observed associations between prenatal PFOS and gross motor functions at 2 years of age, whereas no association was reported between PFOA and any neuro-behavioural outcome [25]. Direct comparison with this study is, however, hampered since their outcome measure was assessed by use of a questionnaire developed specifically for Chinese populations. Some inconsistencies were present in our results of prenatal PFAS exposure and motor development, and we did not observe any consistent and clinically relevant associations which could be due to older age at follow-up or lack of sensitivity of the DCDQ, as the original version of the questionnaire has high sensitivity in populations with high prevalence of motor difficulties and lower sensitivity in a background population [37].

The C8 Health Project (which reports health outcomes in relation to PFOA contamination of drinking water from the Dupont factory in the United States) reported only minor associations between high levels of childhood PFOA measured at 2–8 years of age and a range of neuropsychological outcomes assessed at 6–12 years among a sub-sample of 321 children [38, 39], however, an interaction between PFOA and sex was reported in the analysis of ADHD-like behaviour, indicating high PFOA exposure to be protective among boys and adverse among girls [38]. When *in utero* PFOA exposures were estimated in a pharmacokinetic model, higher PFOA levels were associated with fewer signs of ADHD (using the clinical confidence index of Conners’ continuous performance test- II) [39]. In the study by Stein et al. [38], the exposures were measured postnatally, and in the study by Stein et al., [39], the prenatal exposures were estimated in a toxicokinetic model, whereas we measured the prenatal exposures in maternal blood. Furthermore, different study designs and exposure and outcomes assessments were used in these studies and also, the offspring had different ages at examination, which may explain their findings as we saw no signs of interaction between PFOA and sex.

We acknowledge that this study has some limitations. In the analysis of categorical exposures stratified by country, we used country specific tertiles as exposure levels differed considerably between countries. This could in part explain the inconsistencies in our findings. Furthermore, PFAS levels and SDQ and DCDQ results did vary in the three countries included in this study which further may account for the inconsistent findings between countries.

There is a risk of exposure misclassification since blood samples were collected throughout pregnancy in our study and PFAS tend to decrease during pregnancy [40]. However, we addressed this issue by adjusting for gestational age at blood sampling, although we recognize that this may not have completely eliminated the risk of information bias.

The DCDQ is developed as a screening tool and should not be used for diagnostics [41]. The questionnaire has not been validated in the three countries represented in this study, and thus there is a risk of misclassification of motor problems. However, we believe the questionnaire is suitable for detecting a difference in motor abilities according to PFOS and PFOA exposure levels within the three countries, and since we did not use the cut-offs for indication of motor difficulties, this should not be a large concern.

We chose to use the standard cut-offs for behavioral problems as no country specific cut-offs exist for Greenland, Ukraine and Poland. This enabled us to compare results between countries but may have resulted in misclassification of the SDQ outcome. SDQ as a screening tool has a high validity as well as reliability [42, 43], and our sensitivity analysis, using top ten percentile cut-offs in general suggested consistent results.

Missing data can cause selection bias and to overcome this challenge, we performed several multiple imputation analyses, and results were consistent. A lack of demographic information concerning the Polish non-participants at baseline limited our ability to compare participants and non-participants and selection bias in the Polish results can not be ruled out. Furthermore, the relatively low participation rate of 34% at follow-up in Poland poses a risk of selection bias. However, the non-response analysis at follow-up showed no evidence of difference between responders and non-responders, indicating low risk of selection bias by loss to follow-up.

It is difficult to estimate to what extent postnatal PFAS exposure may have influenced the results. In a sensitivity analysis, we adjusted for duration of breastfeeding, which did not materially change the results. However, residual confounding by postnatal PFAS exposure can not be completely ruled out.

We measured PFAS levels at all periods during pregnancy as a measure of fetal exposure. However, it is not clear to what extent this reflects the vulnerable window of exposure. Others have reported high correlation between 1^st^ and 2^nd^ trimester PFOA levels and because PFOA and PFOS have long half-lives, we believe blood-sampling throughout pregnancy is of minor concern.

The Polish results should be interpreted with caution, since the Polish sample is small. However, samples from Greenland and Ukraine and the pooled analysis are of considerable size and, thus, results should be robust.

## Conclusions

Prenatal exposure to PFOS and PFOA may have a small to moderate effect on children’s neuro-behavioural development at the age of 5–9 years, specifically in terms of hyperactive behaviour. The associations were largest in Greenland, where the exposure contrast is largest. No association was observed in relation to motor skills. Standardized measures of behavioural- and motor problems were used and results were consistent across numerous sensitivity analyses.

## Electronic supplementary material

Additional file 1: Table S1: Associations^a^ between pregnancy levels of PFOS/PFOA (ng/ml) and offspring behavioural problems. Imputation-based results. (DOC 87 KB)

Additional file 2: Table S2: Associations^a^ between pregnancy levels of PFOS/PFOA (ng/ml) and offspring DCDQ-score. Complete-case results. (DOC 43 KB)

Additional file 3: Table S3: Associations^a^ between pregnancy levels of PFOS/PFOA (ng/ml) and offspring SDQ-score. Complete-case results. (DOC 52 KB)

Additional file 4: Table S4: Associations^a^ between pregnancy levels of PFOS/PFOA (ng/ml) and offspring behavioural and hyperactivity problems. Complete-case results. (DOC 57 KB)

Additional file 5: Table S5: Associations^a^ between pregnancy levels of PFOS/PFOA (ng/ml) and offspring behavioural and hyperactivity problems. Imputation-based results. (DOC 58 KB)

## Abbreviations

ADHD: attention deficit hyperactivity disorder
CI: confidence interval
DCDQ: Developmental Coordination Disorder Questionnaire 2007
OR: odds ratio
PFAS: Perfluorinated alkyl substances
PFDoDA: perfluorododecanoic acid
PFNA: perfluorononanoic acid
PFOA: perfluorooctanoate
PFOS: perfluorooctane sulfonate
PFUnDA: perfluoroundecanoic acid
SDQ: Strength and Difficulties Questionnaire
T3: triiodothyronine
T4: total free thyroxine
TSH: thyroid-stimulating hormone.
