# Supplementary material for: Pregnancy serum concentrations of perfluorinated alkyl substances and offspring behaviour and motor development at age 5–9 years – a prospective study
Source: Environ Health. 2015 Jan 7;14:2. doi: 10.1186/1476-069X-14-2 (PMC4298045; doi:10.1186/1476-069X-14-2)
Supplement: Supplementary file 1 — Additional file 1: Table S1: Associationsa between pregnancy levels of PFOS/PFOA (ng/ml) and offspring behavioural problems. Imputation-based results. (DOC 87 KB) [file 12940_2014_821_MOESM1_ESM.doc]

**Table S1 Associations^a^ between pregnancy levels of PFOS/PFOA (ng/ml) and offspring behavioural problems. Imputation-based results**

| **Emotional subscale** | **Combined ^c^, N=1,106**  **Adjusted OR**  **(95 % CI)** | **Greenland, n=526**  **Adjusted OR**  **(95% CI)** | **Ukraine, n=491**  **Adjusted OR**  **(95 % CI)** | **Poland, n=89**  **Adjusted OR**  **(95 % CI)** |
| --- | --- | --- | --- | --- |
| **PFOS** |  |  |  |  |
| Low | 1.0 (ref) | 1.0 (ref) | 1.0 (ref) | - |
| Medium | 0.8 (0.4, 1.9) | 1.1 (0.5, 2.4) | 0.3 (0.1, 0.9) | - |
| High | 0.9 (0.3, 2.6) | 1.0 (0.4, 2.2) | 0.4 (0.1, 1.0) | - |
| Continuous^b^ | 0.9 (0.5, 1.5) | 1.1 (0.5, 2.2) | 0.6 (0.2, 1.3) | - |
| **PFOA** |  |  |  |  |
| Low | 1.0 (ref) | 1.0 (ref) | 1.0 (ref) | - |
| Medium | 1.4 (0.7, 2.7) | 1.7 (0.8, 3.7) | 0.6 (0.2, 1.8) | - |
| High | 1.2 (0.6, 2.7) | 1.0 (0.4, 2.3) | 1.2 (0.5, 3.1) | - |
| Continuous^b^ | 0.9 (0.5, 1.5) | 1.0 (0.5, 2.0) | 0.9 (0.4, 2.0) | - |
| **Conduct subscale** |  |  |  |  |
| **PFOS** |  |  |  |  |
| Low | 1.0 (ref) | 1.0 (ref) | 1.0 (ref) | - |
| Medium | 1.5 (0.8, 2.9) | 1.7 (0.9, 3.3) | 0.6 (0.3, 1.5) | - |
| High | 1.7 (0.7, 4.3) | 0.8 (0.4, 1.7) | 0.8 (0.3, 1.7) | - |
| Continuous^b^ | 1.2 (0.8, 1.9) | 1.0 (0.6, 2.0) | 1.3 (0.6, 2.8) | - |
| **PFOA** |  |  |  |  |
| Low | 1.0 (ref) | 1.0 (ref) | 1.0 (ref) | - |
| Medium | 1.3 (0.7, 2.3) | 1.0 (0.5, 2.0) | 0.8 (0.4, 2.0) | - |
| High | 1.8 (1.0, 3.5) | 1.2 (0.6, 2.3) | 1.6 (0.7, 3.6) | - |
| Continuous^b^ | 1.2 (0.8, 1.9) | 1.3 (0.7, 2.4) | 1.1 (0.8, 1.9) | - |
| **Peer subscale** |  |  |  |  |
| **PFOS** |  |  |  |  |
| Low | 1.0 (ref) | 1.0 (ref) | 1.0 (ref) | - |
| Medium | 1.0 (0.6, 1.7) | 1.2 (0.7, 2.1) | 0.6 (0.3, 1.2) | - |
| High | 1.5 (0.7, 3.0) | 1.6 (0.9, 2.7) | 0.7 (0.4, 1.4) | - |
| Continuous^b^ | 1.2 (0.8, 1.7) | 1.5 (0.9, 2.4) | 0.8 (0.4, 1.3) | - |
| **PFOA** |  |  |  |  |
| Low | 1.0 (ref) | 1.0 (ref) | 1.0 (ref) | - |
| Medium | 1.3 (0.8, 2.0) | 1.9 (0.3, 2.3) | 1.0 (0.5, 1.9) | - |
| High | 1.4 (0.9, 2.3) | 1.1 (0.6, 1.8) | 1.2 (0.6, 2.3) | - |
| Continuous^b^ | 1.1 (0.8, 1.6) | 1.1 (0.7, 1.7) | 1.1 (0.6, 1.8) | - |
| **Pro-social subscale** |  |  |  |  |
| **PFOS** |  |  |  |  |
| Low | 1.0 (ref) | 1.0 (ref) | 1.0 (ref) | - |
| Medium | 1.7 (0.8, 3.5) | 0.8 (0.3, 2.2) | 0.5 (0.2, 1.4) | - |
| High | 1.2 (0.4, 3.8) | 0.5 (0.2, 1.7) | 1.1 (0.4, 2.7) | - |
| Continuous^b^ | 1.1 (0.6, 2.0) | 0.8 (0.3, 2.1) | 1.5 (0.6, 3.7) | - |
| **PFOA** |  |  |  |  |
| Low | 1.0 (ref) | 1.0 (ref) | 1.0 (ref) | - |
| Medium | 1.5 (0.8, 3.0) | 0.4 (0.1, 1.3) | 1.7 (0.6, 5.1) | - |
| High | 0.7 (0.3, 1.7) | 0.4 (0.1, 1.3) | 3.2 (1.1, 9.0)* | - |
| Continuous^b^ | 1.1 (0.6, 2.0) | 0.5 (0.2, 1.3) | 2.0 (1.0, 4.0) | - |

CI, confidence interval; OR, odds ratio; PFOA, perfluorooctanoate acid ; PFOS, perfluorooctane sulfonate; ref, reference group

^a^Adjusted for maternal smoking during pregnancy, maternal alcohol consumption at conception, maternal age at baseline, gestational age at blood sampling and child sex

^b^ The change in OR according to one natural logarithm increase in exposures

^c^ Additionnaly adjusted for country

*Indicates a p-value <0.05
