# Supplementary material for: Pregnancy serum concentrations of perfluorinated alkyl substances and offspring behaviour and motor development at age 5–9 years – a prospective study
Source: Environ Health. 2015 Jan 7;14:2. doi: 10.1186/1476-069X-14-2 (PMC4298045; doi:10.1186/1476-069X-14-2)
Supplement: Supplementary file 2 — Additional file 2: Table S2: Associationsa between pregnancy levels of PFOS/PFOA (ng/ml) and offspring DCDQ-score. Complete-case results. (DOC 43 KB) [file 12940_2014_821_MOESM2_ESM.doc]

**Table S2** **Associations^a^ between pregnancy levels of PFOS/PFOA (ng/ml) and offspring DCDQ-score. Complete-case results**

|  |  | **Combined (N=745)^c^** | | **Greenland (N=217)** | | **Ukraine (N=444)** | | **Poland (N=84)** | |
| --- | --- | --- | --- | --- | --- | --- | --- | --- | --- |
| **DCDQ** |  | **Dif** | **(95 % CI)** | **Dif** | **(95 % CI)** | **Dif** | **(95 % CI)** | **Dif** | **(95 % CI)** |
| **PFOA** | Low | Ref | - | Ref | - | Ref | - | Ref | - |
|  | Medium | -0.6 | -2.1, 0.9 | -1.8 | -4.7, 1.1 | -0.7 | -2.4, 1.0 | 1.4 | -4.6, 7.3 |
|  | High | -0.7 | -2.6, 1.1 | -0.8 | -3.7, 2.1 | -1.1 | -2.9, 0.6 | -4.1 | -10.0, 1.8 |
|  | Continuous^b^ | -0.6 | -1.9, 0.7 | 0.2 | -2.3, 2.7 | -0.4 | -1.8, 1.0 | 3.2 | -9.1, 2.7 |
| **PFOS** | Low | Ref | - | Ref | - | Ref | - | Ref | - |
|  | Medium | -0.8 | -2.4, 0.8 | -0.2 | -2.9, 2.6 | 1.1 | -0.6, 2.8 | -0.6 | -6.6, 5.5 |
|  | High | -0.8 | -3.6, 1.9 | 1.9 | -1.1, 5.0 | 0.0 | -1.8, 1.7 | -2.4 | -8.3, 3.6 |
|  | Continuous^b^ | -0.1 | -1.2, 1.5 | 1.8 | -0.8, 4.5 | -0.1 | -1.6, 1.5 | -2.5 | -9.4, 4.4 |

CI, confidence interval; DCDQ, developmental coordination disorder questionnaire; Dif, difference; PFOA, perfluorooctanoate acid ;

PFOS, perfluorooctane sulfonate; Ref, reference group

^a^ Adjusted for: maternal pregnancy smoking, maternal pre-pregnancy alcohol-intake, maternal age at birth, gestational age at blood-

sampling and child sex

^b^ β= the change in score according to one natural-log unit increase in PFOA and PFOS

^c^ Additionally adjusted for country
