# Supplementary material for: Pregnancy serum concentrations of perfluorinated alkyl substances and offspring behaviour and motor development at age 5–9 years – a prospective study
Source: Environ Health. 2015 Jan 7;14:2. doi: 10.1186/1476-069X-14-2 (PMC4298045; doi:10.1186/1476-069X-14-2)
Supplement: Supplementary file 3 — Additional file 3: Table S3: Associationsa between pregnancy levels of PFOS/PFOA (ng/ml) and offspring SDQ-score. Complete-case results. (DOC 52 KB) [file 12940_2014_821_MOESM3_ESM.doc]

**Table S3 Associations^a^ between pregnancy levels of PFOS/PFOA (ng/ml) and offspring SDQ-score. Complete-case results**

|  |  | | **Combined (n=1,004) ^c^** | | **Greenland (n=468)** | | **Ukraine (n=450)** | | **Poland (n=86)** | |
| --- | --- | --- | --- | --- | --- | --- | --- | --- | --- | --- |
| **Scale** | **Exposure** | | **Dif** | **(95 % CI)** | **Dif** | **(95 % CI)** | **Dif** | **(95 % CI)** | **Dif** | **(95 % CI)** |
| **SDQ** | **PFOA** | Low | Ref | - | Ref | - | Ref | - | Ref | - |
|  |  | Medium | 0.2 | -0.6, 1.0 | 0.3 | -0.8, 1.5 | -0.7 | -1.7, 0.4 | 0.7 | -2.0, 3.4 |
|  |  | High | 0.5 | -0.4, 1.4 | 0.5 | -0.6, 1.6 | -0.2 | -1.3, 0.8 | 1.8 | -0.9, 4.5 |
|  |  | Continuous ^b^ | 0.1 | -0.5, 0.8 | 0.4 | -0.6, 1.5 | -0.4 | -1.2, 0.5 | 2.1 | -0.6, 4.7 |
| **Hyper** |  | Low | Ref | - | Ref | - | Ref | - | Ref | - |
|  |  | Medium | 0.0 | -0.3, 0.4 | 0.4 | -0.1, 0.9 | -0.3 | -0.7, 0.1 | 1.1 | -0.4, 2.6 |
|  |  | High | 0.5 | 0.1, 0.9* | 0.5 | 0.1, 1.0* | -0.2 | -0.6, 0.3 | 0.9 | -0.7, 2.4 |
|  |  | Continuous ^b^ | 0.2 | -0.1, 0.5 | 0.4 | 0.0, 0.9* | -0.1 | -0.5, 0.2 | 1.2 | -0.3, 2.7 |
| **SDQ** | **PFOS** | Low | Ref | - | Ref | - | Ref | - | Ref | - |
|  |  | Medium | -0.2 | -1.1, 0.8 | 1.5 | 0.4, 2.6* | -1.0 | -2.1, 0.0* | -0.2 | -2.9, 2.5 |
|  |  | High | 1.1 | -0.2, 2.4 | 1.2 | 0.1, 2.3* | -1.1 | -2.1, 0.0 | 1.9 | -0.7, 4.6 |
|  |  | Continuous ^b^ | 0.4 | -0.3, 1.0 | 1.1 | 0.1, 2.1* | -0.7 | -1.7, 0.2 | 2.6 | -0.6, 5.7 |
| **Hyper** |  | Low | Ref | - | Ref | - | Ref | - | Ref | - |
|  |  | Medium | 0.1 | -0.3, 0.5 | 0.5 | 0.1, 1.0* | -0.1 | -0.5, 0.3 | 1.0 | -0.6, 2.5 |
|  |  | High | 0.5 | 0.0, 1.1 | 0.4 | -0.1, 0.8 | -0.1 | -0.5, 0.4 | 1.4 | -0.2, 2.9 |
|  |  | Continuous ^b^ | 0.2 | 0.0, 0.5 | 0.3 | -0.1, 0.8 | 0.0 | -0.4, 0.3 | 1.5 | -0.3, 3.2 |

CI, confidence interval; dif, difference; Hyper, hyperactivity sub-scale; PFOA, perfluorooctanoate acid ; PFOS, perfluorooctane sulfonate; Ref, reference group; SDQ, strength and difficulties questionnaire

^a^ Adjusted for: maternal pregnancy smoking, maternal pre-pregnancy alcohol-intake, maternal age at birth, gestational age at blood-sampling and child sex

^b^ β= the change in score according to one natural-log unit increase in PFOA and PFOS

^c^ Additionally adjusted for country

* Indicates a p-value <0.05
