# Supplementary material for: Pregnancy serum concentrations of perfluorinated alkyl substances and offspring behaviour and motor development at age 5–9 years – a prospective study
Source: Environ Health. 2015 Jan 7;14:2. doi: 10.1186/1476-069X-14-2 (PMC4298045; doi:10.1186/1476-069X-14-2)
Supplement: Supplementary file 4 — Additional file 4: Table S4: Associationsa between pregnancy levels of PFOS/PFOA (ng/ml) and offspring behavioural and hyperactivity problems. Complete-case results. (DOC 57 KB) [file 12940_2014_821_MOESM4_ESM.doc]

**Table S4 Associations^a^ between pregnancy levels of PFOS/PFOA (ng/ml) and offspring behavioural and hyperactivity problems. Complete-case results**

| **Scale** | **Cases** | **Combined ^c^**  **Adjusted OR**  **(95 % CI)**  **(N=1,004)** | **Cases** | **Greenland**  **Adjusted OR**  **(95% CI)**  **(n=468)** | **Cases** | **Ukraine**  **Adjusted OR**  **(95 % CI)**  **(n=450)** | **Cases** | **Poland**  **Adjusted OR**  **(95 % CI)**  **(n=84)** |
| --- | --- | --- | --- | --- | --- | --- | --- | --- |
| **SDQ-total** |  |  |  |  |  |  |  |  |
| PFOS |  |  |  |  |  |  |  |  |
| Low | 17 | 1.0 (ref) | 7 | 1.0 (ref) | 10 | 1.0 (ref) | 0 | 1.0 (ref) |
| Medium | 24 | 1.1 (0.5, 2.6) | 13 | 2.4 (0.9, 6.2) | 9 | 0.6 (0.2, 1.6) | 2 | - |
| High | 15 | 1.7 (0.5, 6.0) | 6 | 1.1 (0.3, 3.4) | 6 | 0.5 (0.2, 1.5) | 3 | - |
| Continuous^b^ | 56 | 1.3 (0.7, 2.3) | 26 | 1.1 (0.5, 2.8) | 25 | 1.0 (0.4, 2.6) | 5 | - |
| PFOA |  |  |  |  |  |  |  |  |
| Low | 13 | 1.0 (ref) | 6 | 1.0 (ref) | 7 | 1.0 (ref) | 0 | 1.0 (ref) |
| Medium | 22 | 1.9 (0.9, 4.2) | 10 | 2.6 (0.9, 7.4) | 9 | 1.6 (0.6, 4.6) | 3 | - |
| High | 23 | 2.7 (1.1, 6.5)* | 10 | 2.1 (0.7, 6.1) | 9 | 1.6 (0.6, 4.8) | 2 | - |
| Continuous^b^ | 56 | 1.7 (0.9, 3.0) | 26 | 2.7 (1.0, 7.3)* | 25 | 1.0 (0.4, 2.4) | 5 | - |
| **Hyperactivity** |  |  |  |  |  |  |  |  |
| PFOS |  |  |  |  |  |  |  |  |
| Low | 13 | 1.0 (ref) | 3 | 1.0 (ref) | 7 | 1.0 (ref) | 3 | 1.0 (ref) |
| Medium | 22 | 1.2 (0.6, 2.6) | 8 | 2.8 (0.7, 10.9) | 8 | 1.1 (0.4, 3.2) | 6 | - |
| High | 22 | 2.1 (0.6, 8.2) | 7 | 2.5 (0.6, 10.3) | 9 | 1.4 (0.5, 4.0) | 6 | - |
| Continuous^b^ | 57 | 1.9 (1.0, 3.6) | 18 | 2.3 (0.8, 6.2) | 24 | 1.3 (0.5, 3.5) | 15 | - |
| PFOA |  |  |  |  |  |  |  |  |
| Low | 14 | 1.0 (ref) | 2 | 1.0 (ref) | 9 | 1.0 (ref) | 3 | 1.0 (ref) |
| Medium | 20 | 0.8 (0.3, 1.8) | 7 | 4.2 (0.8, 21.5) | 7 | 0.8 (0.3, 2.3) | 6 | - |
| High | 23 | 2.9 (1.2, 7.0)* | 9 | 6.8 (1.4, 34.0)* | 8 | 0.8 (0.3, 2.3) | 6 | - |
| Continuous^b^ | 57 | 1.6 (0.9, 2.9) | 18 | 5.6 (1.5, 20.6)* | 24 | 0.8 (0.3, 1.9) | 15 | - |

CI, confidence interval; OR, odds ratio; PFOA, perfluorooctanoate acid ; PFOS, perfluorooctane sulfonate; Ref, reference group; SDQ, strength and difficulties questionnaire

* Indicates a p-value <0.05^a^ Adjusted for maternal smoking during pregnancy (serum cotinine ≤10/>10 ng/ml), maternal alcohol consumption at conception (<7/≥7 drinks per week), child sex, maternal age at baseline (continuous) and gestational age at blood sampling (continuous)

^b^ The change in OR according to one natural logarithm increase in exposures

^c^ Additionally adjusted for country
