# Supplementary material for: Pregnancy serum concentrations of perfluorinated alkyl substances and offspring behaviour and motor development at age 5–9 years – a prospective study
Source: Environ Health. 2015 Jan 7;14:2. doi: 10.1186/1476-069X-14-2 (PMC4298045; doi:10.1186/1476-069X-14-2)
Supplement: Supplementary file 5 — Additional file 5: Table S5: Associationsa between pregnancy levels of PFOS/PFOA (ng/ml) and offspring behavioural and hyperactivity problems. Imputation-based results. (DOC 58 KB) [file 12940_2014_821_MOESM5_ESM.doc]

**Table S5** **Associations^a^ between pregnancy levels of PFOS/PFOA (ng/ml) and offspring behavioural and hyperactivity problems. Imputation-based results**

| **Scale**  **Top 10 percentile**  **cut-offs** | **Combined ^e^**  **Adjusted OR**  **(95 % CI)**  **(N=1,106)** | **Greenland**  **Adjusted OR**  **(95% CI)**  **(n=526)** | **Ukraine**  **Adjusted OR**  **(95 % CI)**  **(n=491)** | **Poland**  **Adjusted OR**  **(95 % CI)**  **(n=89)** |
| --- | --- | --- | --- | --- |
| **SDQ-total^b^** |  |  |  |  |
| PFOS |  |  |  |  |
| Low | 1.0 (ref.) | 1.0 (ref.) | 1.0 (ref.) | 1.0 (ref.) |
| Medium | 0.7 (0.4, 1.4) | 2.1 (0.9, 4.6) | 0.4 (0.2, 0.9) | - |
| High | 1.2 (0.5, 3.2) | 1.3 (0.5, 3.0) | 0.6 (0.2, 0.9) | - |
| Continuous^d^ | 0.9 (0.6, 1.5) | 1.2 (0.6, 2.4) | 0.5 (0.3, 1.0) | - |
| PFOA |  |  |  |  |
| Low | 1.0 (ref.) | 1.0 (ref.) | 1.0 (ref.) | 1.0 (ref.) |
| Medium | 1.4 (0.8, 2.5) | 2.0 (0.9, 4.5) | 0.7 (0.3, 1.6) | - |
| High | 2.3 (1.2, 4.5) | 2.2 (0.9, 5.0) | 1.1 (0.5, 2.2) | - |
| Continuous^d^ | 1.6 (1.0, 2.4) | 2.2 (1.0, 4.7) | 0.9 (0.5, 1.7) | - |
| **Hyperactivity^c^** |  |  |  |  |
| PFOS |  |  |  |  |
| Low | 1.0 (ref.) | 1.0 (ref.) | 1.0 (ref.) | 1.0 (ref.) |
| Medium | 0.9 (0.5, 1.5) | 1.6 (0.7, 3.8) | 0.9 (0.5, 1.7) | - |
| High | 1.3 (0.5, 3.3) | 1.4 (0.6, 3.4) | 0.7 (0.4, 1.4) | - |
| Continuous^d^ | 1.1 (0.7, 1.7) | 1.4 (0.7, 3.0) | 0.8 (0.4, 1.4) | - |
| PFOA |  |  |  |  |
| Low | 1.0 (ref.) | 1.0 (ref.) | 1.0 (ref.) | 1.0 (ref.) |
| Medium | 1.1 (0.6, 1.8) | 2.4 (0.9, 6.4) | 0.7 (0.4, 1.5) | - |
| High | 1.7 (0.9, 3.2) | 3.0 (1.2, 7.9) | 0.8 (0.4, 1.6) | - |
| Continuous^d^ | 1.2 (0.8, 1.8) | 2.6 (1.1, 6.1) | 0.7 (0.4, 1.2) | - |

CI, confidence interval; OR, odds ratio; PFOA, perfluorooctanoate; PFOS, perfluorooctane sulfonate; SDQ, strength and difficulties questionnaire

^a^Adjusted for maternal cotinine level during pregnancy, maternal alcohol consumption at conception, child sex, maternal age at pregnancy and gestational age at blood sampling

^b^SDQ cut-offs: normal and borderline (0 to 16) versus abnormal (16 to 40)

^c^Hyperactivity cut-offs: normal and borderline (0 to 6) versus (6 to 10)

^d^The change in OR according to one natural logarithm increase in exposures

^e^ Additionally adjusted for country
